# Structural factors of benzylated glucopyranans for shear-induced adhesion†

**DOI:** 10.1039/c9ra02644d

**Published:** 2019-08-21

**Authors:** Abu Bin Ihsan, Yuta Kawaguchi, Hiromichi Inokuchi, Hiroshi Endo, Yasuhito Koyama

**Affiliations:** Department of Pharmaceutical Engineering, Faculty of Engineering, Toyama Prefectural University 5180 Kurokawa Imizu Toyama 939-0398 Japan ykoyama@pu-toyama.ac.jp; Department of Mechanical Systems Engineering, Faculty of Engineering, Toyama Prefectural University 5180 Kurokawa Imizu Toyama 939-0398 Japan

## Abstract

We have prepared benzylated glucopyranans and evaluated the structural effects on the adhesion capacity. It was found that 97%-benzylated (1→2)-glucopyranan exhibited a unique shear-induced adhesion. The effects of structural factors on the adhesion behaviors are discussed through systematic adhesion tests, differential scanning calorimetry, theoretical models, and IR spectroscopy.

## Abstract 2

Shear-induced adhesion of benzylated (1→2)-glucopyranan is attributed to the kinetically stabilized elongated structure and the switchable projection of hydroxyl groups induced by stress.

## Graphical abstract

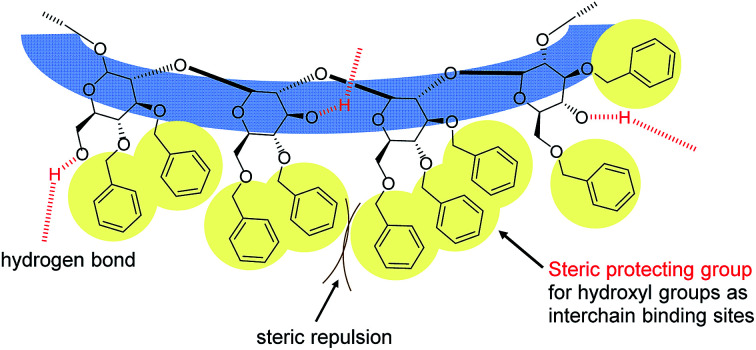

The development of new bioglues has been strongly urged from the viewpoint of advancing biomedical engineering.^1–4^ The shear-induced adhesion behaviors of von Willebrand factor (VWF)^5–7^ is a nice motif to create a new bioglue. VWF is a mosaic plasma protein. At the sites of vascular injury, shear and elongated flows of fluid alter the original coiled structure of VWF to an elongated structure. The shear-induced structural change of VWF exposes the multiple binding sites of VWF to receptors on platelets and collagen, which remarkably increases the adhesion of VWF, resulting in hemostasis or thrombosis. It is noted that the original coiled structure of VWF hardly exhibits adhesive properties. Apart from the adhesion mechanism of VWF, such shear-induced adhesion analogous to that of VWF should be actively studied as the function of bioglues, which could provide unprecedented bioglues with mechanical responsivity, stress-resistance, and readhesion capacity in the future. However, the structure of VWF comprising the permutation of 20 natural amino acids is too complicated to generalize the shear-induced adhesion behavior.

Based on this background, we have focused on the simple structures of glucopyranans (Glcps) and their potential applicability to adhesive materials possessing shear-induced adhesion. Glcp is a member of polysaccharides and consists of glucose as a repeating unit, whose structure should be simpler than that of VWF. Gelatinized starch has been long used as an adhesive material for paper products and food additives.^8–11^ Amylose as a Glcp bearing 1,4-glycosidic bonds [Glcp-(1-4)] is the ingredient of starch (Fig. 1a). The adhesion of amylose is densely related to the formation of hydrogen bonds. Conversely, the complete protection of hydroxyl groups in amylose can prevent the adhesion. Thus, we envisioned the preparation of nearly preprotected amylose as a candidate for the shear-responsive adhesive material, strongly assuming the following idea: if the mechanical stress to the polymer exposes the interchain binding sites, such as hydrogen bonding exposed, the polymer should serendipitously show an increase in the adhesive strength (*S*_Adh_) after applying stress. To assess the postulation, we primarily prepared benzylated Glcp-(1-4) as the glue. Moreover, we have recently reported a new synthetic method of glycosides grafting (1→2)-glucopyranan [Glcp-(1-2)] (Fig. 1b).^12^ Since a series of synthetic Glcp-(1-2) derivatives was available in our group, we also prepared benzylated Glcp-(1-2)-*X* with different benzylation ratios (*X*%) as the additional candidate of the glue.

**Fig. 1:**
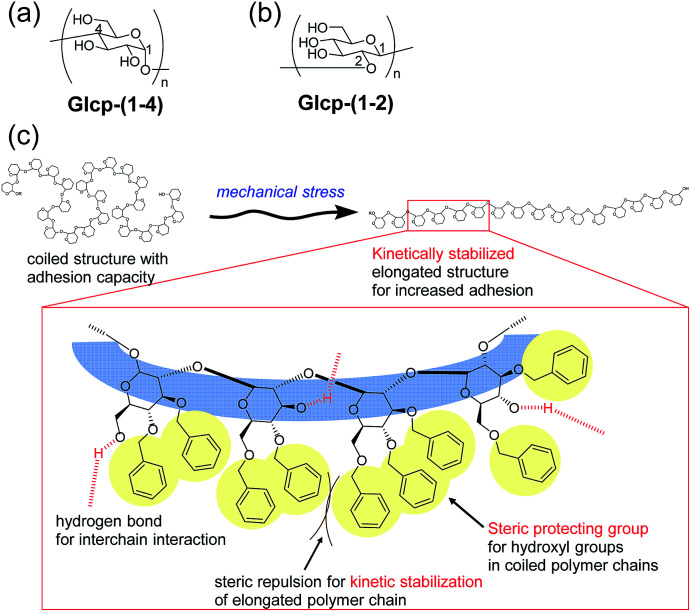
(a) Structures of Glcp-(1-4) and (b) Glcp-(1-2) and (c) the plausible structure of the kinetically stabilized elongated chain of adhesive Glcp-(1-2)-*X*.

Herein, we investigated lap-shear tests using two glass plates with Glcp-based glues to evaluate the effects of the structure, stretching velocity (*v*), shear modulus (*G*), and water content on *S*_Adh_, as well as the shear-induced adhesion behavior. We found that Glcp-(1-2)-97 exhibited a unique shear-induced adhesion. However, the polymer as casted on the glass hardly exhibited adhesive properties, the uniaxial orientation of the polymer by shear stress afforded a remarkable increase in *S*_Adh_. It was indicated that the benzyl groups of Glcp-(1-2)-97 appeared to be steric protecting groups not only to hinder the alcohols in the original coiled structure but also to kinetically stabilize the shear-induced elongated polymer structure (Fig. 1c).

Benzylated Glcp-(1-4) was prepared by treating amylose (synthetic, *M*_n_ 31 kDa) with benzyl chloride and powdered NaOH in DMSO according to the literature.^13^ By the ^1^H NMR spectrum, the benzylation ratio was determined to be 95%.^14^ We performed differential scanning calorimetry (DSC) measurements on the polymers. The glass transition temperature (*T*_g_) of Glcp-(1-4)-95 was found to be 23.7 °C.^14^ With the obtained Glcp-(1-4)-95 in hand, we performed lap-shear tests using two glass plates with Glcp-(1-4)-95 as a glue to clarify the adhesion behaviors. The polymer was homogeneously coated on a glass plate with an area of 20 × 25 mm, and another glass plate was attached to the polymer-coated area (Fig. 2a). Both ends of the glass plates were clamped to a tensile machine. Initially, shear adhesive tests on the polymer samples were performed at a stretching velocity of 10 mm min^−1^. However, even after the repeated application of stress to the glue, Glcp-(1-4)-95 showed negligible adhesion (0.05 kPa), implying that the original structure of Glcp-(1-4)-95 might be rigid and sufficiently stable, which may hardly expose interchain binding sites after applying mechanical stress.

**Fig. 2:**
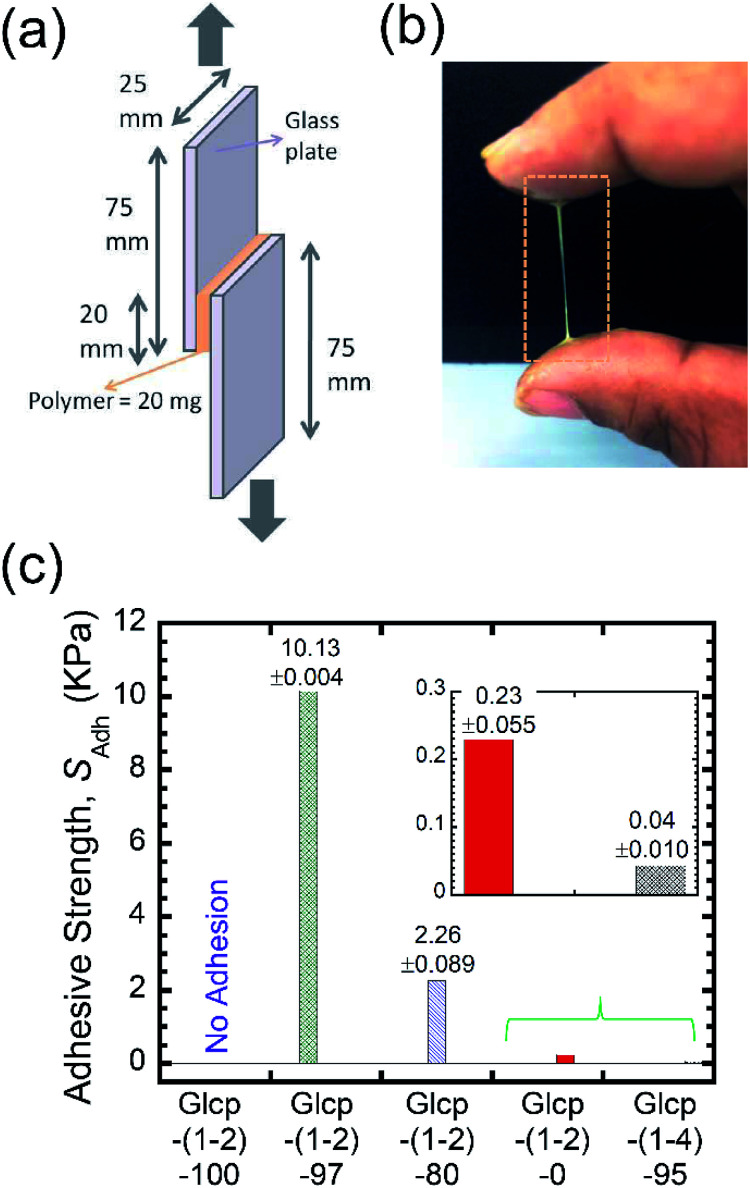
(a) Adhesion testing geometry to measure *S*_Adh_, (b) a photograph of the macroscopic fiber formation of Glcp-(1-2)-97, and (c) the dependence of *S*_Adh_ on the benzylation ratio. Bars are average values from three samples. The relative standard deviations (%RSD) are 0.04% for Glcp-(1-2)-97, 3.9% for Glcp-(1-2)-80, 24% for Glcp-(1-2)-0, and 23% for Glcp-(1-4)-95.

Thus, we prepared Glcp-(1-2)-based adhesives, as shown in Scheme 1, because the main chain can be regarded as a functionalized polyoxyethylene that seems to be more flexible than that of amylose. We selected 4-penten-1-ol as an initiator, because the terminal olefinic proton signals in the ^1^H NMR spectrum would be a diagnostic tool to estimate the degree of polymerization (DP) and the number-average molecular weight (*M*_n_). Sugar-based cyclic sulfite (1) was treated with a catalytic amount of trifluoromethanesulfonic acid (TfOH) in the presence of 4-penten-1-ol (2 mol%) and molecular sieves of 3Å (MS 3A) in CH_2_Cl_2_ at room temperature.^12^ The required reaction time was 7 d for the completion of reaction. The structure of the obtained polymer [Glcp-(1-2)-100] was confirmed by IR, ^1^H NMR, and ^13^C NMR spectra.^14^ The IR spectrum of Glcp-(1-2)-100 does not include the absorption signal attributed to the sulfite linkage at around 1200 cm^−1^,^14^ suggesting the perfect elimination of SO_2_ from the main chain. From the integral ratio between the internal olefin and the aromatic proton signals in the ^1^H NMR spectrum,^14^ we estimated the DP and *M*_n_ to be 51.2 and 22 kDa, respectively. The results indicate that the polymerization degree is controllable by the feed ratio of the initiator. The anomeric stereochemistry was almost β (>95%), which was estimated by the ^13^C NMR spectrum using the integral ratio between the carbon signals at 110–100 ppm for β carbons and those at 100–90 ppm for α carbons,^14^ implying that the cationic chain propagation proceeded in an inversion manner *via* the successive pseudo S_N_2 reactions of alcohol at the anomeric centers. To obtain Glcp-(1-2)-*X* with different benzylation ratios, we next performed the hydrogenolysis of Glcp-(1-2)-100 using Pd(OH)_2_ as a catalyst. The mixture was stirred for different reaction intervals under H_2_ atmosphere to furnish three types of polymers with 97, 80, and 0% benzylation ratios, where the benzylation ratio was determined by the integral ratio between the aromatic proton signals at 6.8–7.8 ppm and the aliphatic proton signals at 3.0–5.5 ppm in the ^1^H NMR spectra. The glass transition temperatures (*T*_g_) were −5.6 °C for Glcp-(1-2)-100, 13.7 °C for Glcp-(1-2)-97, 19.3 °C for Glcp-(1-2)-80, and 29.8 °C for Glcp-(1-2)-80.^14^ These results suggest that the benzyl protection makes the polymer chain plasticized and benzylated Glcp-(1-2) is more flexible than benzylated Glcp-(1-4).

**Scheme 1:**
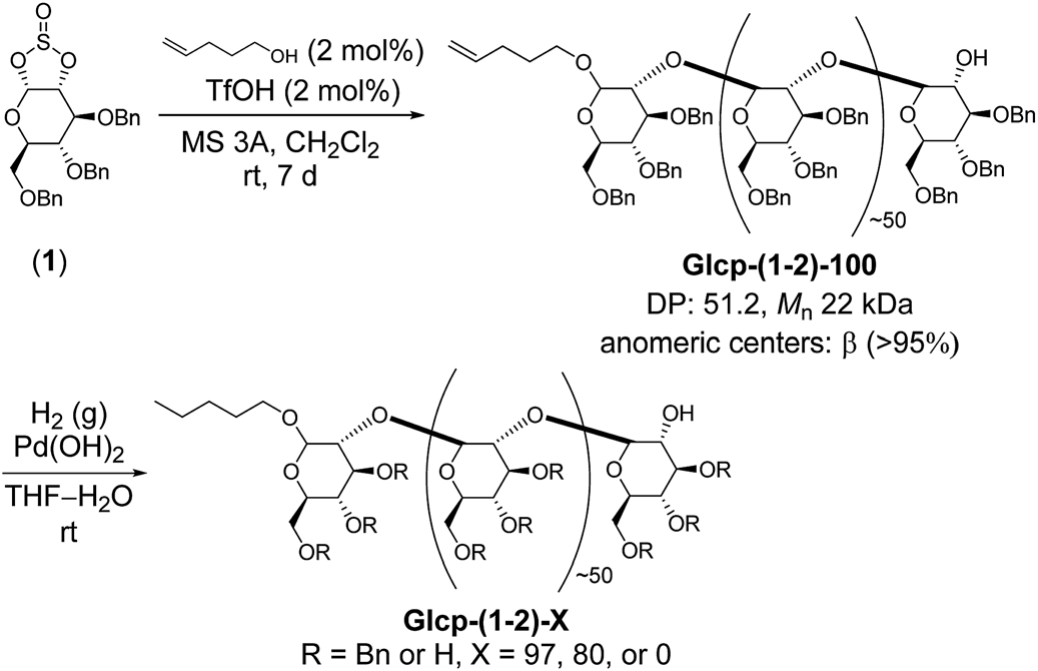
Synthesis of Glcp-(1-2)-based adhesives. Glcp-(1-2) with *X*% benzylation is abbreviated as Glcp-(1-2)-*X*.

We performed the lap-shear tests of all Glcp-(1-2) derivatives using three samples to obtain the average values. As a result, it turned out that the order of *S*_Adh_ was Glcp-(1-2)-80 (2.3 kPa) > Glcp-(1-2)-97 (0.8 kPa) > Glcp-(1-2)-0 (0.3 kPa), while for Glcp-(1-2)-100 was not possible to measure *S*_Adh_. In order to confirm the readhesion capacity of the polymers, the detached glass plates were joined again by pressing them slightly with a finger for 30 s at room temperature after the first measurement (Cycle 1), followed immediately by the lap-shear test for subsequent cycles [Cycles 2–6, Fig. 3a (green filled circles) and ESI Fig. S10a†].^14^ Surprisingly, the *S*_Adh_ of Glcp-(1-2)-97 increased remarkably from the 1^st^ to 5^th^ cycle and then saturated at around 10 kPa, which is stronger than the reported value for fibrin glue (7 kPa).^4^ The results are in a good contrast to that of Glcp-(1-4)-95 with a similar benzylation ratio, clearly suggesting the impact of the glycosidic position on the adhesion capacity of Glcps. After applying stress, Glcp-(1-2)-97 was also found to show a highly viscoelastic nature, which easily formed a fiber (Fig. 2b). Moreover, the *S*_Adh_ values of Glcp-(1-2)-80 and Glcp-(1-2)-0 hardly changed after the repeated tests. Fig. 2c summarizes the adhesibility results in terms of the saturated *S*_Adh_ values after the repeated tests. These results suggest that the *S*_Adh_ values increase along with the increase in the benzylation ratio, expect for Glcp-(1-2)-100, seem to be correlated to the *T*_g_ values. To know the effects of hydrogen bonds on *S*_Adh_, the glass plate coating Glcp-(1-2) was completely dried at 90 °C under reduced pressure for 24 h and tested for repeated adhesion capacity. The obtained *S*_Adh_ of the dried adhesive was quite high at Cycle 1, which reached approximately 263 ± 12 kPa but decreased with the repeated tests (Fig. 3a, green open circles). The tendency of the repeated tests appears to be opposite to the wet polymer, indicating not only the reinforcement of adhesion based on the efficient interchain hydrogen bonding but also the role of water as a plasticizer. Moreover, the *S*_Adh_ value of Glcp-(1-2)-80 was not related to the repeated cycle.

**Fig. 3:**
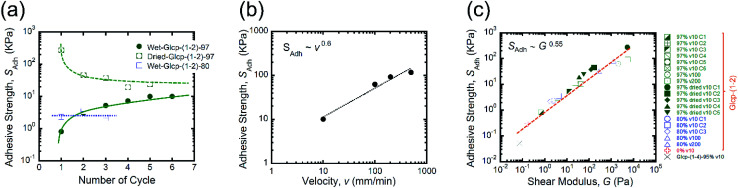
(a) Dependence of *S*_Adh_ on the number of cycles by the repeated lap-shear tests of Glcp-(1-2)-*X* using the same sample (*v*: 10 mm min^−1^), (b) dependence of the *S*_Adh_ value of Glcp-(1-2)-97 on *v*, and (c) the proportional relationship of *S*_Adh_ to *G*^0.55^. Lines are to guide the eyes.

In order to understand the effects of *v* on the adhesion, we subsequently investigated the dependency of *S*_Adh_ of Glcp-(1-2)-97 on *v* (Fig. 3b and ESI Fig. S10b and c†).^14^ The adhesion of Glcp-(1-2)-97 between the glass plates was clearly sensitive to *v*. The *S*_Adh_ values are plotted as a function of *v*, which is described by a power-law expression. Consequently, it turned out that the *S*_Adh_ values were correlated to *v* to the power of 0.6. According to the theoretical prediction by Schallamach,^15^ the adhesion force between ideal elastomers would correlate to *v* in a power-law manner with an exponent of 2/3. Thus, the experimental exponent value indicates that the adhesive properties of Glcp-(1-2)-*X* would almost coincide with those of an elastomer. We also evaluated the correlations between *S*_Adh_ and *G* for all tested samples (Fig. 3c), considering that the Chernyak–Leonov model for the friction of viscoelastic materials anticipates a proportional relationship of *S*_Adh_ to *G*^0.5^.^16–18^ All plots appear to be straight lines, regardless of different structures and *v*. The *G* values for all plots are below 10^5^ Pa, which are within the empirical criterion for adhesive materials proposed by Dahlquist.^19–21^ The approximate straight line indicates that *S*_Adh_ is correlated to *G* to the power of 0.55, which agrees with the theoretical Chernyak–Leonov model. The results obtained from Fig. 3b and c provide evidence for the high viscoelasticity of Glcp-(1-2)-97 as well as the efficient energy dissipation capacity of Glcp-(1-2)-97 on adhesion.

We will now organize the observed adhesive behaviors of Glcp-based adhesives. Based on the low *T*_g_ and the mechanical responsivity of Glcp-(1-2)-97 as an ideal elastomer, the coiled polymer structure of Glcp-(1-2)-97 in the virgin adhesive would uniaxially align in the tensile direction to give the elongated structure during the lap-shear tests as expected in Fig. 1c. The rigid and bulky repeating structure of Glcp-(1-2)-97 may kinetically suppress the structural relaxation due to the steric repulsion. In the cases of Glcp-(1-2)-80 and Glcp-(1-2)-0, the increased *T*_g_s would make the mechanical responsivity weaken and these less bulky repeating units would hardly prevent the reformation of the original coiled structure. It is highlighted that the elongated structure of Glcp-(1-2)-97 shows a remarkably high *S*_Adh_ than the virgin polymer as a coiled structure. The shear-induced adhesive change in Glcp-(1-2)-97 seems to be similar to that of VWF in blood as described in the introduction. In a similar manner to VWF, the elongated polymer chain of Glcp-(1-2)-97 is expected to expose the hydrogen bonds as intermolecular binding sites, leading to an increase in *S*_Adh_. It is interesting that Glcp-(1-2)-97 exhibits the strongest *S*_Adh_ after applying stress in spite of the least content of hydroxyl groups in the polymers. The results imply that after applying stress the hydroxyl groups in Glcp-(1-2)-97 would facilitate the interchain hydrogen bond formations as physical cross-links. To evaluate the special hydrogen bonding capacity in Glcp-(1-2)-97, we compared the IR spectra of the polymers (Fig. 4). In the spectra of both Glcp-(1-2)-80 and Glcp-(1-4)-95, the absorbance signals of the hydroxyl groups appear at around 3300 cm^−1^, which indicate the strong hydrogen bonding of the hydroxyl groups in these polymers.^22^ Moreover, the signal of virgin Glcp-(1-2)-97 appears at 3427 cm^−1^, which could be assigned to free OH.^22^ This means that the hydroxyl groups in virgin Glcp-(1-2)-97 would hardly contribute to the intra- and intermolecular interactions. The exposed hydroxyl groups after applying stress would facilitate interchain hydrogen bonding, which should render a strong *S*_Adh_ based on the formation of a physical network structure. Moreover, the hydroxyl groups in virgin Glcp-(1-2)-80 and Glcp-(1-4)-95 would form stable intramolecular hydrogen bonds against the mechanical stress probably at the hydrophilic domains, which may suppress the interchain binding of the hydroxyl groups. The IR absorbance wavelength of hydroxyl groups in Glcp derivatives seems to be a diagnostic tool to judge the active binding sites (OH) to the shear-induced adhesion. It is highlighted that the benzyl groups in the polymer appear to be steric protecting groups not only to kinetically stabilize the shear-induced elongated polymer structure but also to hinder the hydroxyl groups in the original coiled structure.

**Fig. 4:**
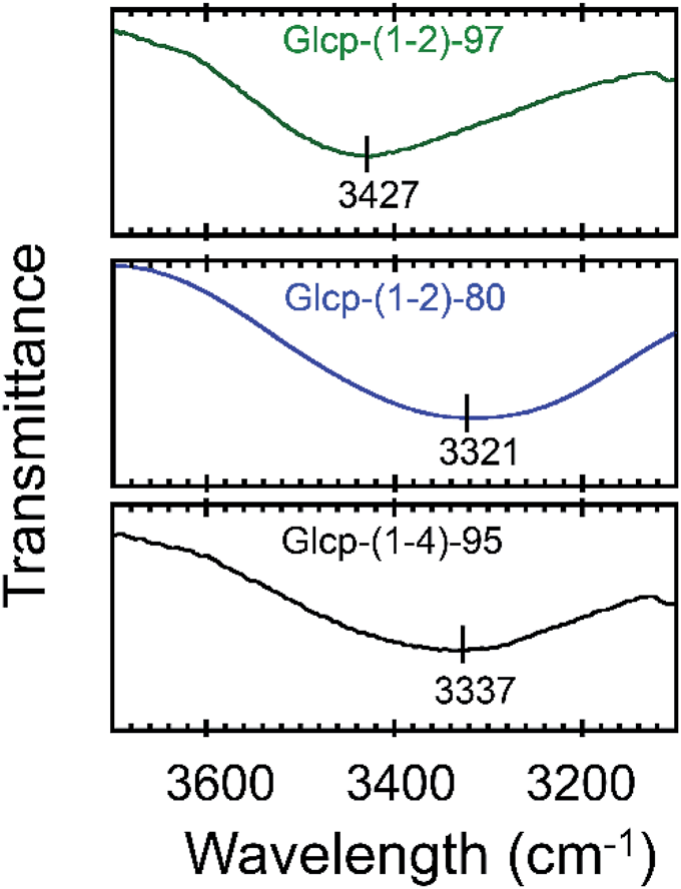
Partial IR spectra of Glcp-(1-2)-97, Glcp-(1-2)-80, and Glcp-(1-4)-95.

In conclusion, we developed a new class of adhesive materials comprising benzylated Glcp-(1-2) skeletons. We evaluated the important structural factors on the shear-induced adhesion, such as kinetically stabilized elongated structure and switchable projection of hydroxyl groups induced by stress. It was found that Glcp-(1-2)-97 exhibited optimal adhesive properties, such as higher *S*_Adh_ than that of conventional fibrin glue and a readhesion capacity. The effects of *v* and *G* on *S*_Adh_ revealed that the adhesion properties of Glcp-(1-2)-97 between glass plates almost coincided with those of an ideal elastomer, implying that Glcp-(1-2)-97 had a strong energy dissipation capacity on adhesion. These systematic experiments in this study should motivate synthesis chemists to produce shear-induced adhesive materials based on the other rational combinations of polysaccharides with functional groups. As polysaccharides are predicted to be nontoxic, biodegradable, and biocompatible according to the backbone,^23–25^ this concept of designing adhesive materials will be useful for creating polysaccharide-based stimuli-responsive bioglues in the near future.

## Conflicts of interest

There are no conflicts to declare.

## Supplementary Material

RA-009-C9RA02644D-s001
