# Supplementary material for: Structural factors of benzylated glucopyranans for shear-induced adhesion
Source: RSC Adv. 2019 Aug 21;9(45):26214–8. doi: 10.1039/c9ra02644d (PMC9070370; doi:10.1039/c9ra02644d)
Supplement: RA-009-C9RA02644D-s001 [file RA-009-C9RA02644D-s001.pdf]

Electronic Supplementary Material (ESI) for RSC Advances.
This journal is © The Royal Society of Chemistry 2019

Electronic Supplementary Materials

Structural Factors of Benzylated Glucopyranans for Shear-Induced Adhesion

Abu Bin Ihsan,[a] Yuta Kawaguchi,[b] Hiromichi Inokuchi,[b] Hiroshi Endo,[b]

Yasuhito Koyama*[a]

[a]Department of Pharmaceutical Engineering, Faculty of Engineering
Toyama Prefectural University, 5180 Kurokawa, Imizu, Toyama 939-0398, Japan
[b]Department of Mechanical Systems Engineering, Faculty of Engineering
Toyama Prefectural University, 5180 Kurokawa, Imizu, Toyama 939-0398, Japan
*E-mail: ykoyama@pu-toyama.ac.jp

Contents

General methods **2**

Typical procedure for the deprotection of benzyl group **3**

Synthesis of benzylated amylose **3**

^{1}H NMR, ^{13}C NMR, and IR spectra **4**

Lap shear testing **9**

Lap shear test results **11**

DSC profiles **12**

General methods:

Materials. Dehydrated dichloromethane (Kanto Chemical Co., Inc., Tokyo, Japan), trifluoromethanesulfonic acid (TfOH, Kanto Chemical Co., Inc. , Tokyo, Japan), 4-penten-1-ol (Tokyo Chemical Industry Co., Inc. , Tokyo, Japan), 50 wt% $Pd(OH)_2$ (Tokyo Chemical Industry Co., Inc., Tokyo, Japan), tetrahydrofuran (THF, Kanto Chemical Co., Inc., Tokyo, Japan), amylose (M_n 31,200, Glico Nutrition Co., Ltd., Osaka, Japan), dimethylsulfoxide (DMSO, Tokyo Chemical Industry Co., Inc., Tokyo, Japan), powdered sodium hydroxide (NaOH, Sigma-Aldrich, Darmstadt, Germany), and benzyl chloride (Tokyo Chemical Industry Co., Inc., Tokyo, Japan) were used without further purification. Granular molecular sieves 3Å (MS 3A) as a dehydrator was activated by careful heating by a heat gun under vacuum. The other chemicals were used without purification. Sugar-based cyclic sulfite was prepared according to the literature: S. S. Sangeetha, Y. Koyama, *Tetrahedron Lett.,* 2016, **57**, 3657. Perbenzylated amylose was prepared according to the literature: J. N. Bemiller, R. E. Wing, *Carbohydr. Res.*, 1968, **6**, 197.

Measurements. ^{1}H NMR (400 MHz) and ^{13}C NMR (100 MHz) spectra were recorded on Bruker AVANCE II 400 spectrometers (Brucker, Fällanden, Switzerland) using $CDCl_3$ and DMSO as the solvent, calibrated using an internal standard and residual undeuterated solvent signals. In the cases of ^{1}H NMR spectra in DMSO-d_6, a small amount of CF_3COOH was added to the NMR sample in order to shift the water signal. FT-IR spectra via an attenuated total reflection (ATR) method were measured using a Perkin Elmer spectrum 100 spectrometer (Perkin Elmer, Shelton, USA). Differential scanning calorimetry (DSC) analyses were carried out on DSC-60 plus (Shimadzu Co. Ltd., Kyoto, Japan) for polymers under N_2 atmosphere (flow rate: 50 mL/min). The lap-shear adhesive test was performed by the tensile machine EZ-SX (Shimadzu Co. Ltd., Kyoto, Japan) at a different shear velocity.

Typical procedure for the deprotection of benzyl group

The solution of benzylated glucopyranan (**Glcp-(1-2)-100**, 1.1 g) in THF (60 mL) was added to the suspension of $Pd(OH)_2$ (50 wt%, 0.55 g) in water (30 mL). The reaction system was degassed 3 times using vacuum pump and the atmosphere was replaced to H_2. The mixture was stirred for different time interval under H_2 atmosphere. The reaction mixture was filtered through a celite pad and the cake was repeatedly washed with MeOH. The filtrate was concentrated in vacuo and dried under vacuum to give the partially deprotected polymer.

Synthesis of benzylated amylose

Amylose (100 mg, M_n 31,200) and DMSO (4.0 mL) were placed in a round-bottomed flask under Ar atmosphere. The flask was placed in an oil bath at 60 °C. The mixture was stirred until the complete dissolution of amylose for 1 h and cooled to room temperature. To the mixture was added finely powdered sodium hydroxide (NaOH, 672 mg) and additional DMSO (2.0 mL). This mixture was stirred for 1 h at the same temperature. Benzyl chloride (0.78 mL) was added dropwise to the mixture. The mixture was warmed to 70 °C and stirred for 16 h. After 1, 2, and 16 h, an additional 0.2 mL of benzyl chloride was added dropwise. After 16 h, the mixture was warmed to 85 °C and further stirred for 2 h. The mixture was cooled to room temperature, and 10 ml of water and 10 ml of chloroform were added in that order. The chloroform layer was removed, and the aqueous layer was extracted with chloroform for three times. The chloroform extracts were combined, washed successively with 30 mL of water, 20 mL of 0.5 M sulfuric acid, and 40 mL of water, and evaporated under diminished pressure at 60 °C to syrup. The resulting crude was reprecipitated into MeOH. The precipitates were collected by filtration and dried in vacuo to give benzylated amylose [**Glcp(1-4)-95**] as a white solid in a quantitative yield.

^1H NMR, ^{13}C NMR, and IR spectra

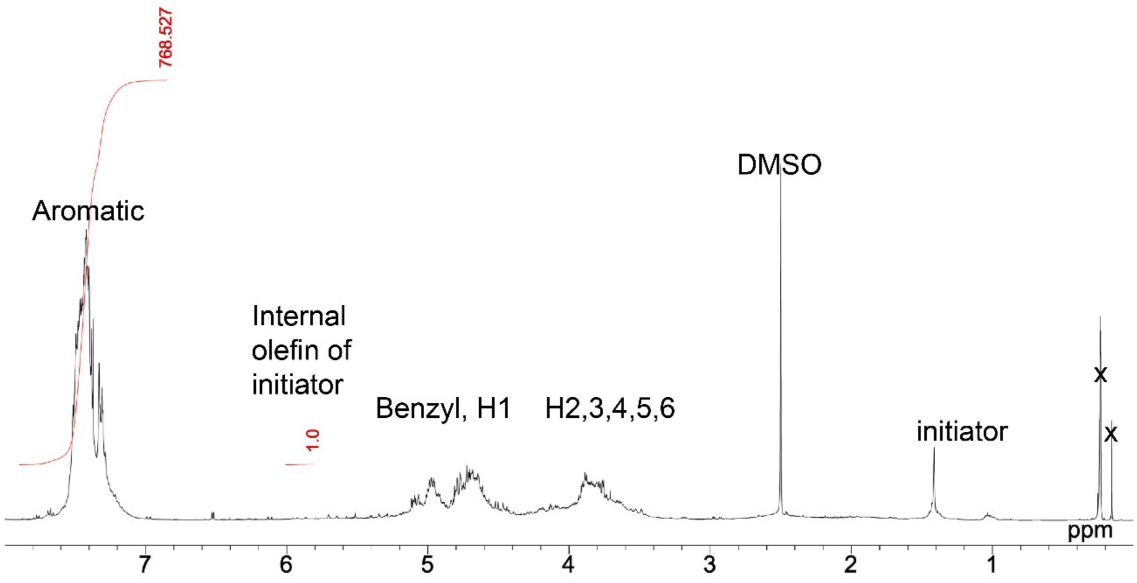

Figure S1: ^1H NMR spectrum of **Glcp-(1-2)-100** (400 MHz, DMSO-d_6 with a small amount of CF_3COOH, 298 K).

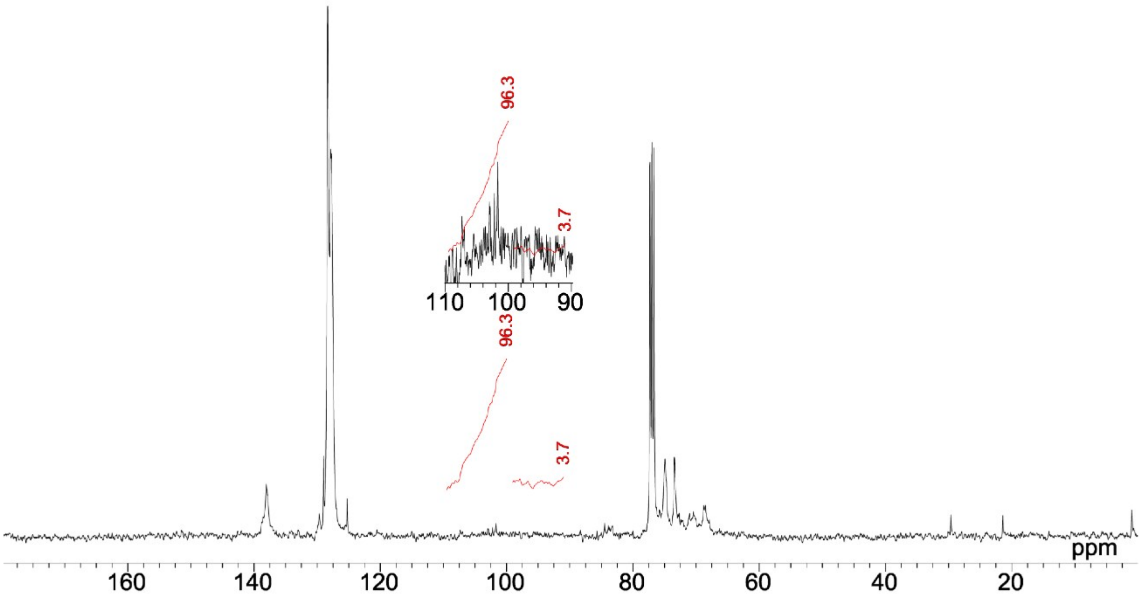

Figure S2: ^{13}C NMR spectrum of **Glcp-(1-2)-100** (100 MHz, $CDCl_3$, 298 K) and the inset of expanded anomeric carbon region.

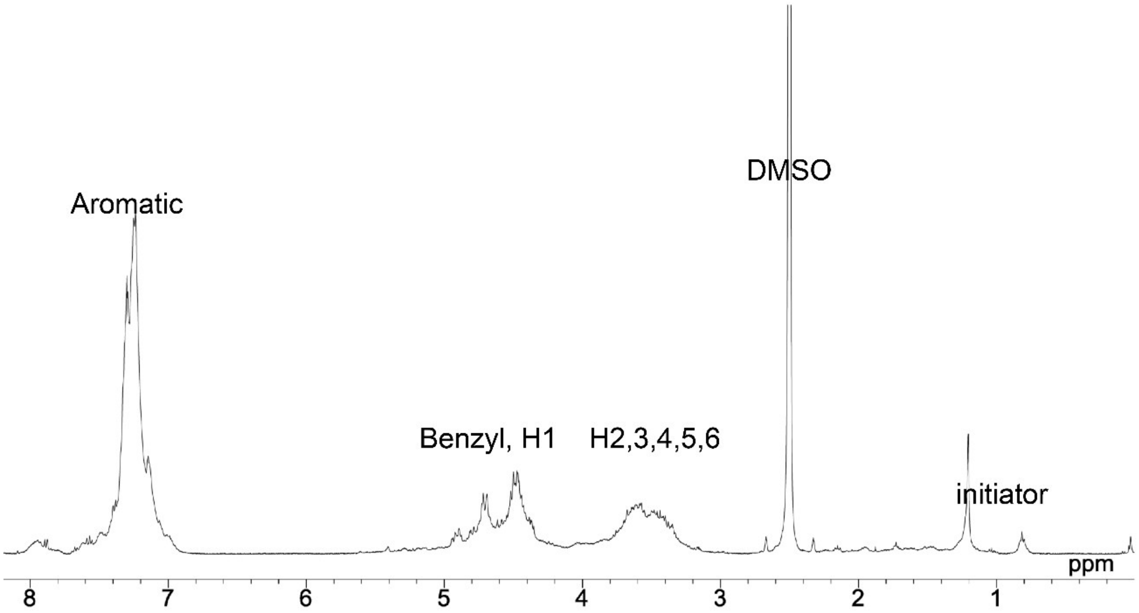

Figure S3: ^{1}H NMR spectrum of **Glcp-(1-2)-97** (400 MHz, DMSO-d_6 with a small amount of CF_3COOH, 298 K).

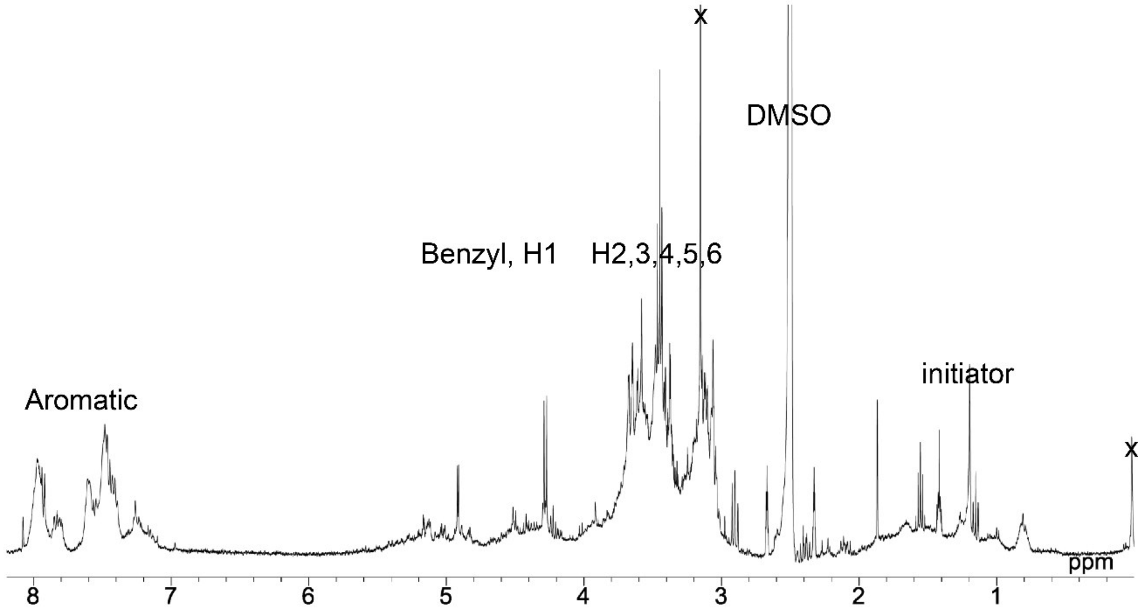

Figure S4: ^{1}H NMR spectrum of **Glcp-(1-2)-80** (400 MHz, DMSO-d_6 with a small amount of CF_3COOH, 298 K).

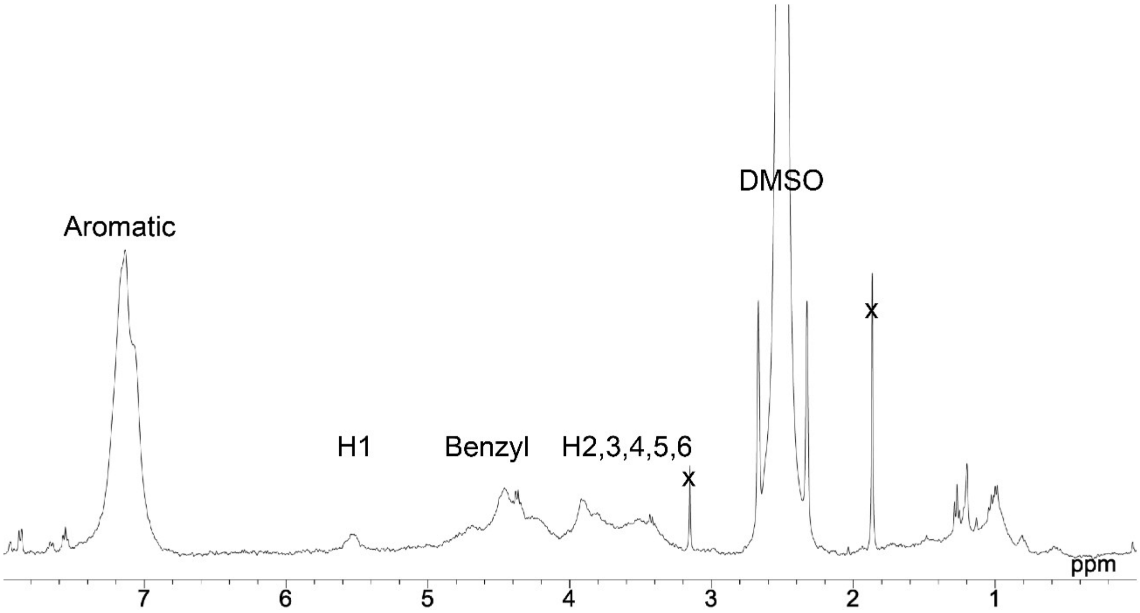

Figure S5: ^{1}H NMR spectrum of **Glcp-(1-4)-95** (400 MHz, DMSO-d_6 with a small amount of CF_3COOH, 298 K).

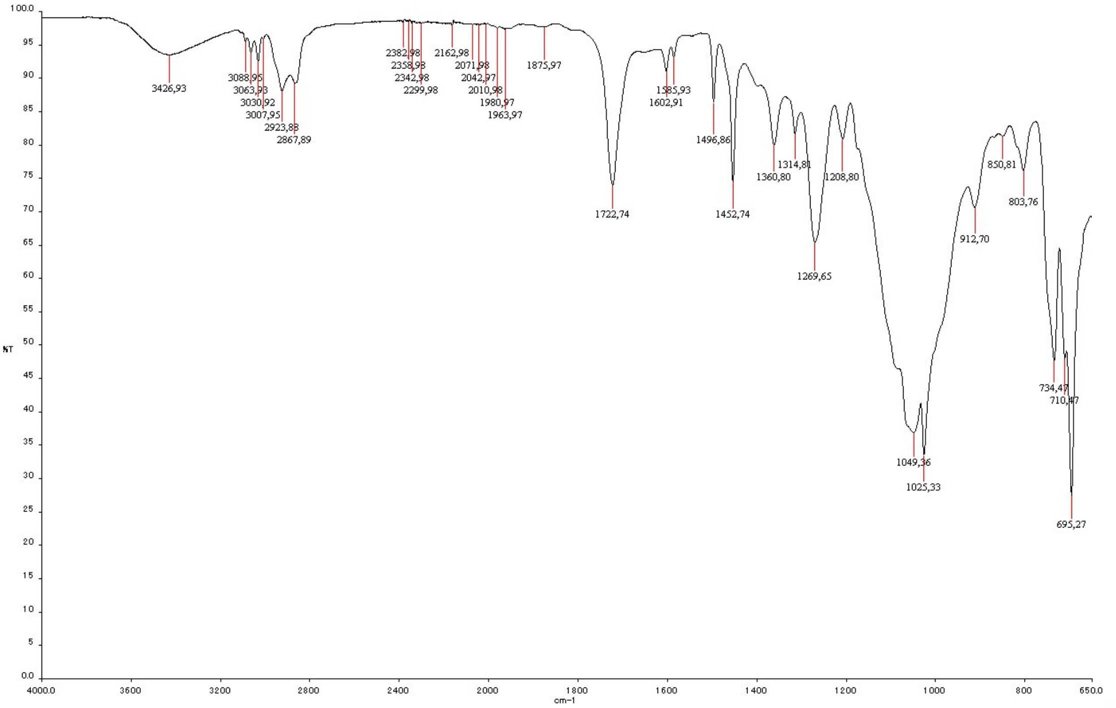

Figure S6. IR spectrum of **Glcp-(1-2)-97** (ATR).

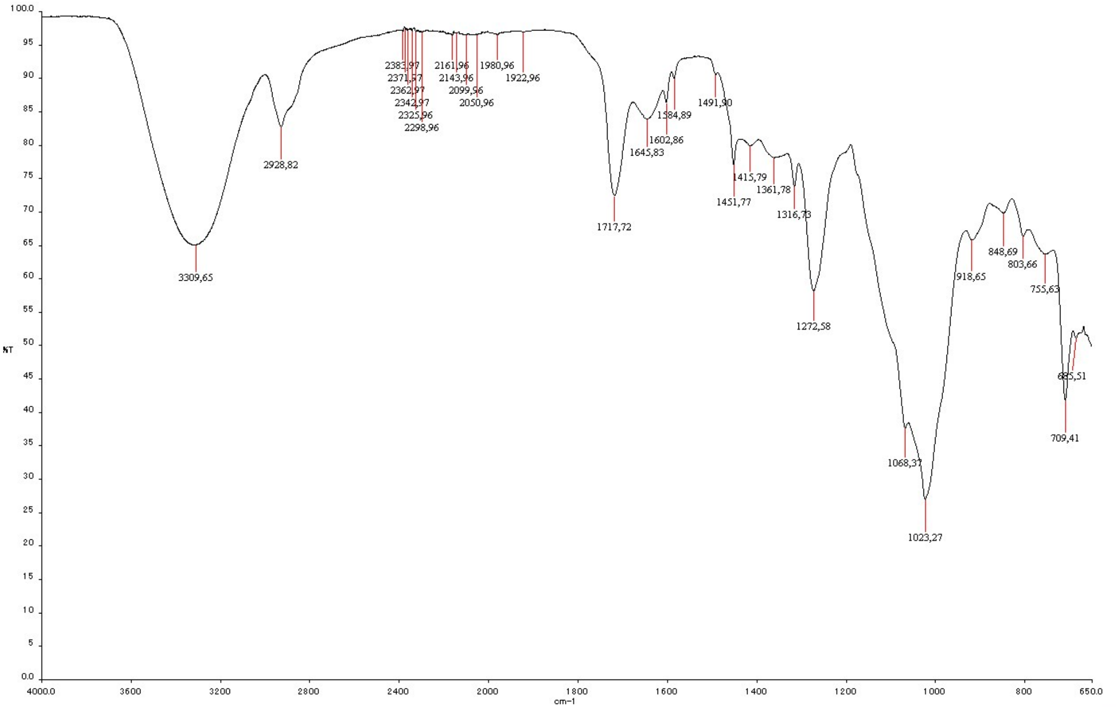

Figure S7. IR spectrum of **Glcp-(1-2)-80** (ATR).

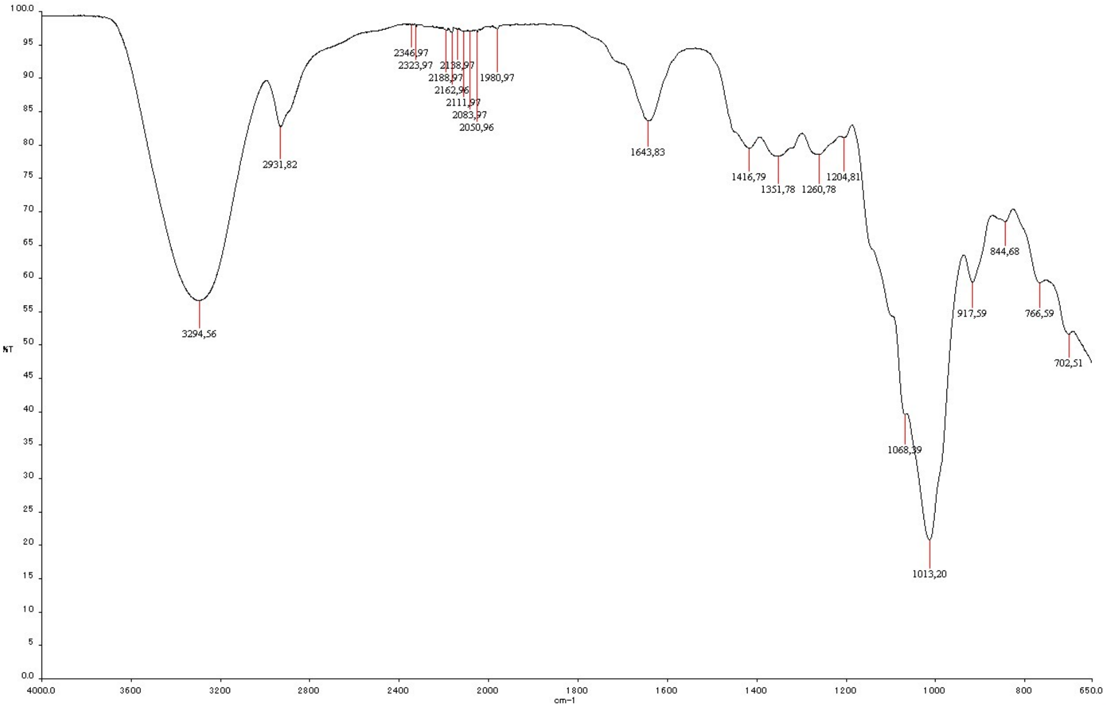

Figure S8. IR spectrum of **Glcp-(1-2)-0** (ATR).

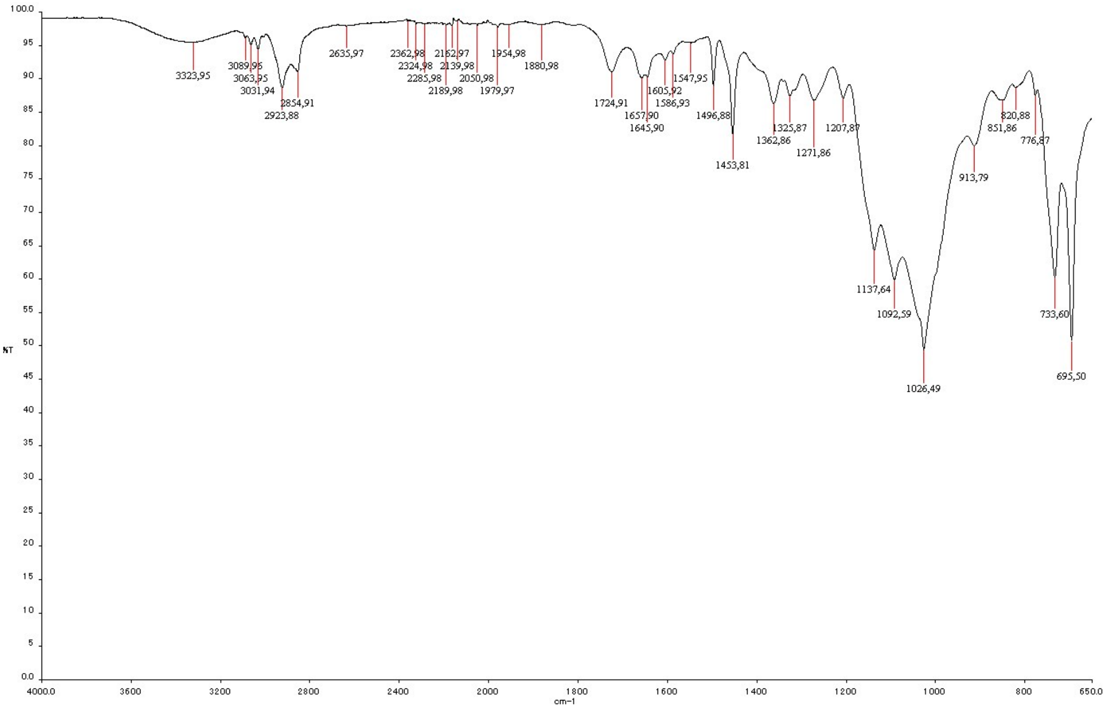

Figure S9. IR spectrum of **Glcp-(1-4)-95** (ATR).

Lap-shear testing

Two glass plates (length × width = 75 mm × 25 mm) were brought into contact with a fixed quantity (20 mg) of polymers, which formed a junction contact area of 5.0 cm^2 ($l_0 \times w$ = 20 mm × 25 mm). The lap joint was slightly pressurized with a finger for 30 s before clamping the two ends of the glass plate to a tensile machine (EZ-SX; Shimadzu Co., Ltd.). Shear adhesive tests were performed at a different shear velocity (v). S_{Adh}, defined as the strength of the lap-shear sample immediately before the interface of the two adhered surfaces begins to separate, was estimated from the measured maximum adhesive force (F_{max}) according to the following expression: $S_{Adh} = F_{max}$/surface ($l_0 \times w$), where $l_0 \times w$ = 20 mm × 25 mm. G was obtained by dividing S_{Adh} by the strain: $G = S_{Adh}/(l/l_0)$. The area and thickness of glue at the lap joint were constant during the re-adhesion experiments.

Lap shear test results

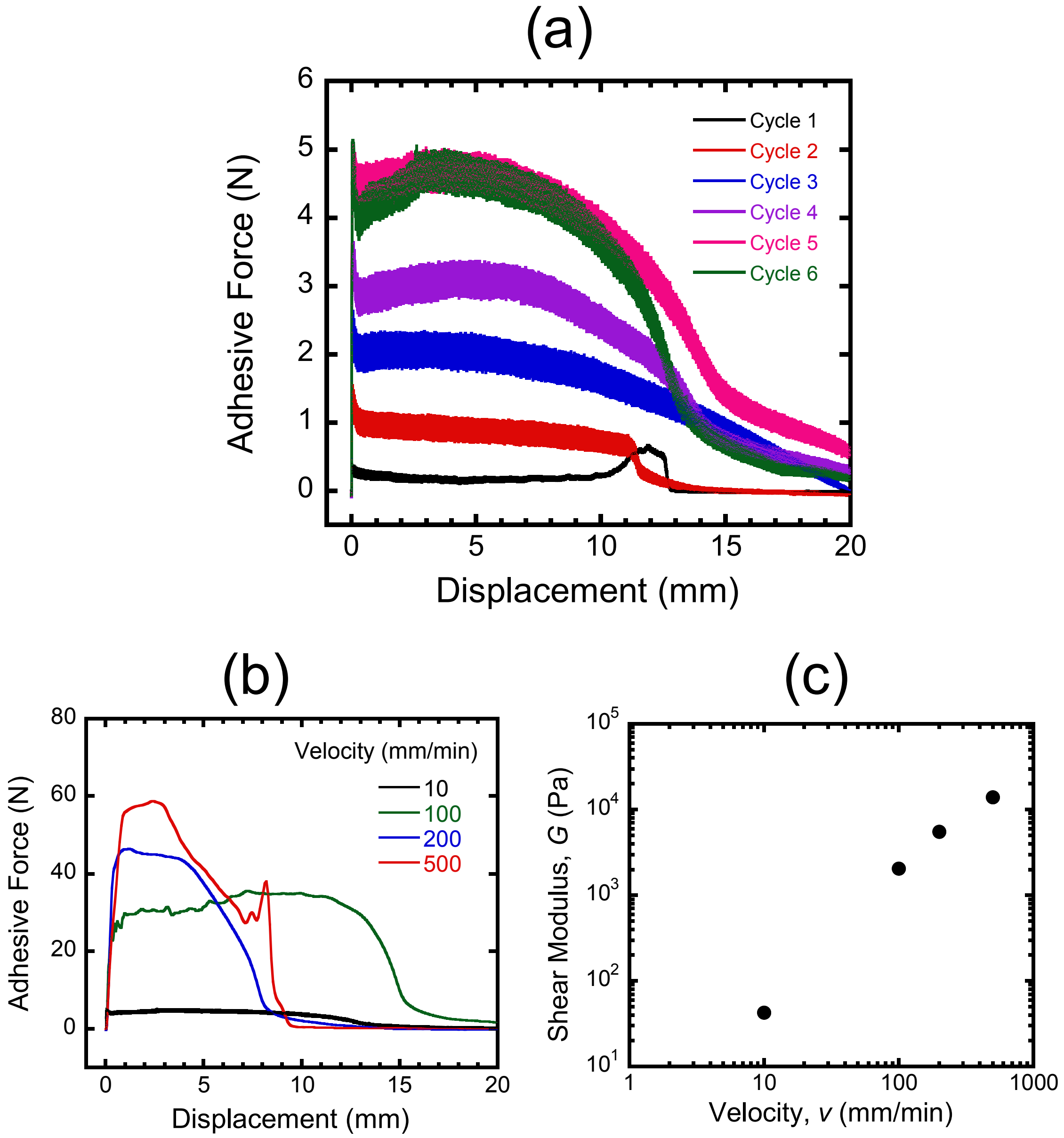

Figure S10. Adhesive behaviors of **Glcp-(1-2)-97**. (a) Repeated lap-shear tests of **Glcp-(1-2)-97** at a stretching velocity (v) of 10 mm/min using the same sample on glass, (b) force–displacement curves from the lap-shear test to measure the adhesion of alternating peptides to glass at different stretching velocities, and (c) dependence of v on the modulus (G).

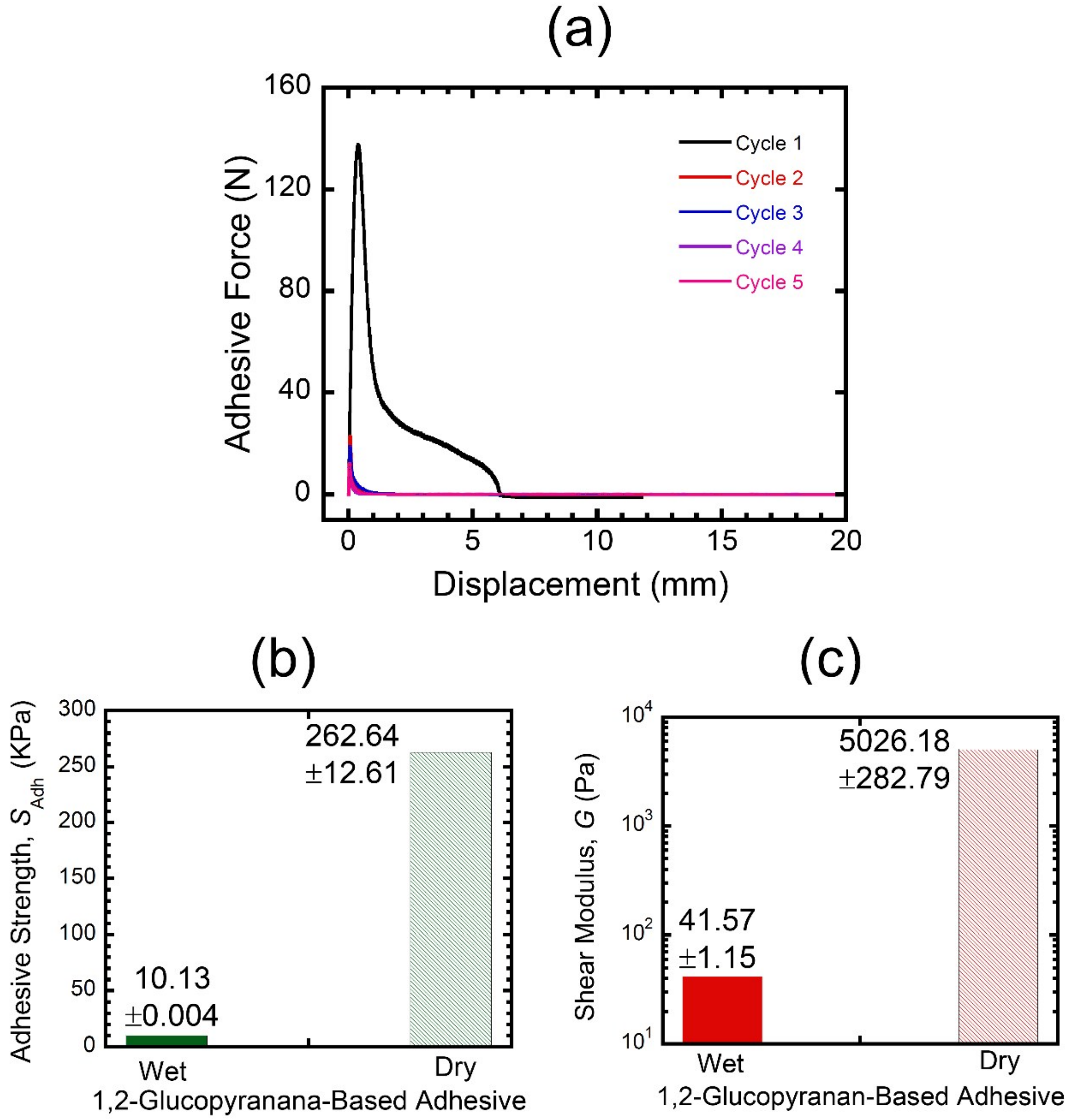

Figure S11. Adhesive behaviors of dried **Glcp-(1-2)-97**. (a) Repeated lap-shear tests of dried **Glcp-(1-2)-97** at a stretching velocity (v) of 10 mm/min using the same sample on glass, (b) dependence of adhesive strength (S_{adh}) on drying, and (c) comparative modulus (G) on two different state. Bars are average values from three samples. The relative standard deviations (%RSD) are (b) 0.04% for wetted and 4.8% for dried and (c) 2.8% for wetted and 5.6% for dried.

DSC profiles

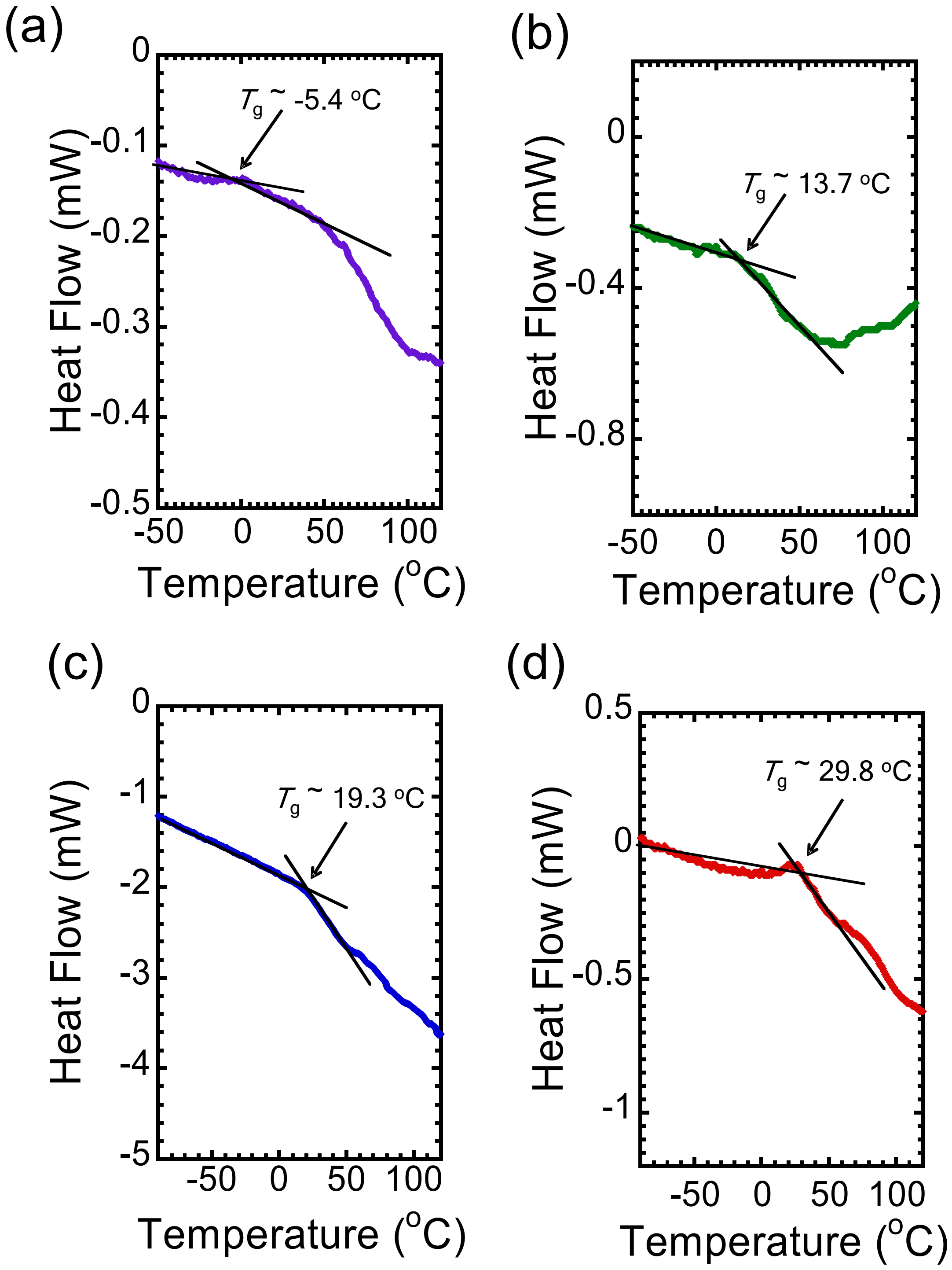

Figure S12. DSC profiles of 1,2-glucopyranan-based adhesives with different benzylation ratio (a) **Glcp-(1-2)-100**, (b) **Glcp-(1-2)-97**, (c) **Glcp-(1-2)-80**, and (d) **Glcp-(1-2)-0** (10 °C/min).

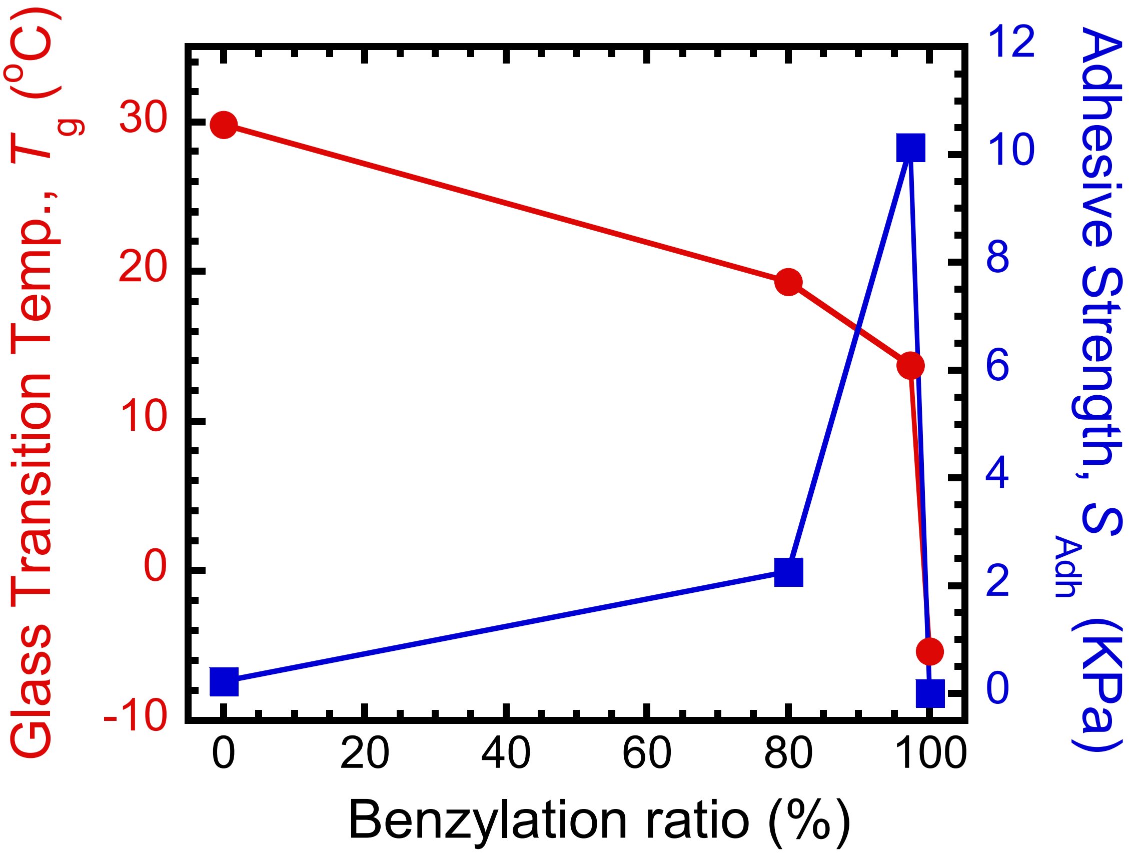

Figure S13. Dependence of adhesive strength (S_{adh}) and glass transition temperatures on different benzylation ratio of 1,2-glucopyranan-based adhesives.

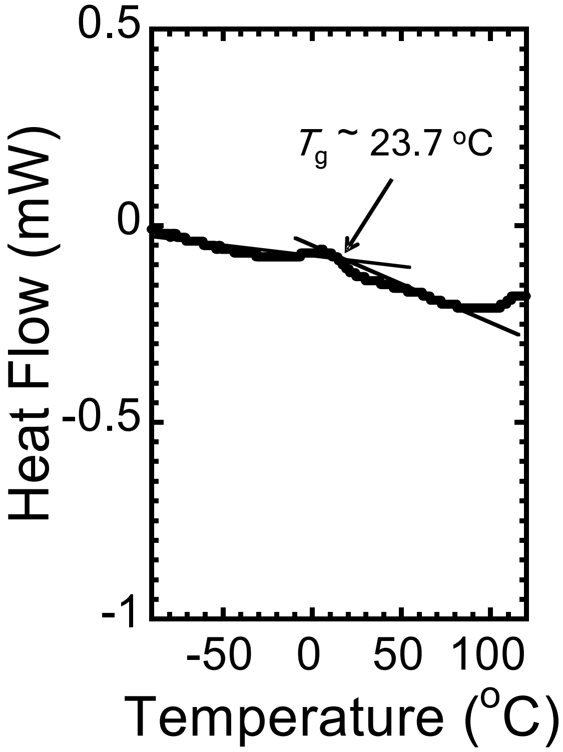

Figure S14. DSC profiles of **Glcp-(1-4)-95** (10 °C/min).
